# Modelled and observed mean and seasonal relationships between climate, population density and malaria indicators in Cameroon

**DOI:** 10.1186/s12936-019-2991-8

**Published:** 2019-11-10

**Authors:** Amelie D. Mbouna, Adrian M. Tompkins, Andre Lenouo, Ernest O. Asare, Edmund I. Yamba, Clement Tchawoua

**Affiliations:** 1 Laboratory for Environmental Modelling and Atmospheric Physics (LEMAP), Department of Physics, Faculty of Science, University of Yaoundé́ I, Yaoundé, Cameroon; 2 Earth System Physics, Abdus Salam International Centre for Theoretical Physics (ICTP), Strada Costiera 11, Trieste, Italy; 3 Department of Physics, Faculty of Science, University of Douala, Douala, Cameroon; 4 Department of Epidemiology of Microbial Diseases, Yale School of Public Health, Yale University, New Haven, USA; 5 Department of Physics, Kwame Nkrumah University of Science and Technology, Kumasi, Ghana

**Keywords:** Malaria, Climate, Cameroon, Parasite ratio, Entomological inoculation rate

## Abstract

**Background:**

A major health burden in Cameroon is malaria, a disease that is sensitive to climate, environment and socio-economic conditions, but whose precise relationship with these drivers is still uncertain. An improved understanding of the relationship between the disease and its drivers, and the ability to represent these relationships in dynamic disease models, would allow such models to contribute to health mitigation and adaptation planning. This work collects surveys of malaria parasite ratio and entomological inoculation rate and examines their relationship with temperature, rainfall, population density in Cameroon and uses this analysis to evaluate a climate sensitive mathematical model of malaria transmission.

**Methods:**

Co-located, climate and population data is compared to the results of 103 surveys of parasite ratio (PR) covering 18,011 people in Cameroon. A limited set of campaigns which collected year-long field-surveys of the entomological inoculation rate (EIR) are examined to determine the seasonality of disease transmission, three of the study locations are close to the Sanaga and Mefou rivers while others are not close to any permanent water feature. Climate-driven simulations of the VECTRI malaria model are evaluated with this analysis.

**Results:**

The analysis of the model results shows the PR peaking at temperatures of approximately 22 °C to 26 °C, in line with recent work that has suggested a cooler peak temperature relative to the established literature, and at precipitation rates at 7 mm day^−1^, somewhat higher than earlier estimates. The malaria model is able to reproduce this broad behaviour, although the peak occurs at slightly higher temperatures than observed, while the PR peaks at a much lower rainfall rate of 2 mm day^−1^. Transmission tends to be high in rural and peri-urban relative to urban centres in both model and observations, although the model is oversensitive to population which could be due to the neglect of population movements, and differences in hydrological conditions, housing quality and access to healthcare. The EIR follows the seasonal rainfall with a lag of 1 to 2 months, and is well reproduced by the model, while in three locations near permanent rivers the annual cycle of malaria transmission is out of phase with rainfall and the model fails.

**Conclusion:**

Malaria prevalence is maximum at temperatures of 24 to 26 °C in Cameroon and rainfall rates of approximately 4 to 6 mm day^−1^. The broad relationships are reproduced in a malaria model although prevalence is highest at a lower rainfall maximum of 2 mm day^−1^. In locations far from water bodies malaria transmission seasonality closely follows that of rainfall with a lag of 1 to 2 months, also reproduced by the model, but in locations close to a seasonal river the seasonality of malaria transmission is reversed due to pooling in the transmission to the dry season, which the model fails to capture.

## Background

Malaria is a life-threatening disease caused by parasites that are transmitted through the bites of infected mosquitoes [1]. Globally the disease is present and endemic in tropical regions where the climate and hydrological conditions are suitable for the vector survival and development of the parasite. In Cameroon, malaria has always been and still remains a major health problem [2]. It is a major endemic illness and the leading cause of morbidity and mortality in the country. Children aged 0 to 5 and pregnant women are the most vulnerable category with a total of 22% of morbidity and mortality risk [3, 4]. Moreover the 2000–2010 national health report precise that the disease was responsible for medical consultation (40–45%), morbidity (50%), deaths in children under five (40%), deaths in health institutions (30 to 40%), days spent in hospital (57%) and sick leave (26%) in the country [2, 5].

Intervention strategies have recently been increased by the national programme to fight malaria, in the form of free distribution of insecticide-treated mosquito nets (ITNs) and free consultation and treatment of uncomplicated malaria in children under 5 years [2]. The high incidence of malaria in Cameroon is not surprising due to the presence of the three key vectors: namely *Anopheles gambiae*, *Anopheles funestus* and *Anopheles arabiensis* across the country [6, 7]. In terms of species distribution, Hamadou et al. [8] found that *An. gambiae* alone accounts for 90%, with the remaining 10% made up of *An. funestus* and *An. arabiensis*.

As in other sub-Saharan African countries [9–13], there is a spatio-temporal variation in malaria transmission across ecological zones in Cameroon (namely, the Soudano-Sahelian zone, the Adamaoua plateau, the Savannah-forest, the south equatorial forest, the western plateau and the costal zone [14]). The peak transmission period is related to the key periods of rainfall with a delay of 1 or 2 months for the vector/parasite cycles to amplify, as temperatures are usually within the range that support both mosquito survival and parasite development [15, 16]. During the monsoon season, temporary transient ponds and puddles become abundant, and can serves as potential breeding habitats for malaria vectors [11]. Temperatures are important for regulating the intensity of transmission however, as they impact the life cycles and mortalities rate of the vector as well as the sporogonic cycle of the parasite [17].

While the broad relationships between climate and malaria transmission are broadly under-stood, the exact nature of is still uncertain. Regarding the temperature relationship, earlier work [17] suggested that falciparum transmission increased above a threshold of approximately 18 °C to peak at a temperature of around 28 to 32 °C, decreasing thereafter due to the higher mortality of the adult vector. Ermert et al. [18] highlighted the large uncertainty of vector mortality at warm temperatures, while more recently, incorporation of new data and knowledge of the temperature sensitivity larvae stages of the vector has led to the suggestion that the transmission peak in fact occurs at considerably cooler temperatures [19–21].

In view of this uncertainty, the first aim of this work is to relate the malaria prevalence as measured by the parasite ratio (PR) gathered from a large number of field surveys to the mean climate in each locations in the months preceding the field survey, using data mostly gathered in the period before the large scale up of interventions. While such an analysis can reveal broad time-averaged relationships between malaria and climate, it cannot inform on the seasonality of the disease. Firstly, the prevalence is a time-integrated metric of the disease due to slow natural clearance times, with immune individuals often having low background parasite counts continuously in endemic areas [22, 23], and additionally field PR surveys are isolated in time. A better metric for seasonality is the transmission rate, as measured by the entomological inoculation rate (EIR), the number of infective bites per person per unit time. A newly released database of EIR is thus utilized [24], which contains year-long records of monthly EIR measurements in order to be able to examine the seasonality of disease transmission in Cameroon.

Many previous studies have shown how vicinity to breeding sites could be a key determinant of hazard of exposure to the disease [25–28], but few have studied how water proximity may alter the seasonality of disease transmission. Away from permanent water bodies, one expects the disease transmission to track the occurrence of seasonal rains closely, as these provide the temporary breeding sites preferred by the vector *An. gambiae* [29, 30], but with a temperature-determined delay of 1 to 2 months due to the “spin-up” amplification of the vector and parasite life cycles [27, 31]. Vicinity to breeding sites that may form near the edges of permanent water bodies, such as lakes, may reduce the seasonal variation of transmission, or may even reverse the relationship altogether in the case of river systems that are either intermittent or perennial but subject to large seasonal flow variations, and that may form large-scale pooling during their transition to the dry season [32].

In addition to climate, differences in population density contribute to the observed variability in malaria transmission intensity between rural, peri-urban and urban settings [33], due to land use patterns, density of households, access to social and health services and the dilution effect [34]. Thus, analysis are also made on how population density may influence the malaria diagnostics. If the climate and population link to malaria can be represented in dynamical models [35–37], these models can act as useful tools to understand how climate trends, extreme seasonal anomalies or variability associated with, for example, the El Nino southern oscillation, may potentially affect transmission and such models could possibly be used for mitigation or adaptation decision support. The second aim of this paper is to use the malaria-climate-population analysis to evaluate gridded simulations of malaria transmission made with dynamical malaria model that accounts for both population density and climate.

## Methods

### Study area and climate data

**Fig. 1:**
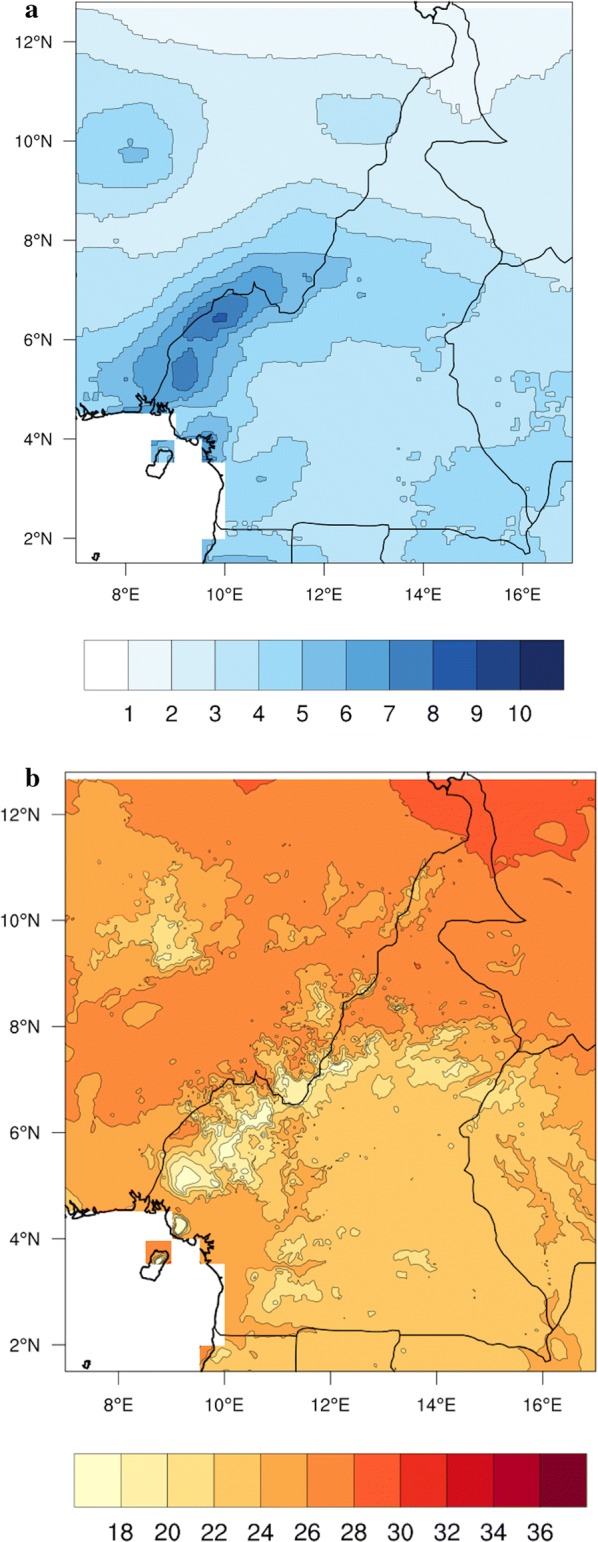
Map of Cameroon and neighbouring countries showing mean rainfall and temperature from 1985 to 2006. **a** Rainfall (mm/day); **b** temperature (°C)

The study is conducted in Cameroon situated in central Africa within 1.5–13° N and 8–17° E with others neighbouring countries (Fig. 1). The country climate is influenced by the Harmattan and the Atlantic Monsoon winds. Cameroon is characterized by two climatic domains: the tropical climatic domain that stretches to the north, extending into the Sahel zone (~ 8° to 13° N) [38, 39] and the humid equatorial domain that covers the rest of the country (~ 1.5° to 8° N).

The equatorial domain is characterized by heavy rainfall events, with increasing temperatures and a degrading vegetation as one moves far from the Equator [40]. It presents two rainy seasons with abundant rainfall that can reach 2200 mm year^−1^ and two dry seasons with average temperature of 25° C [41]. The tropical area, which is usually recognized with high temperatures (up to 33 °C) and low rainfall (maximum of 1500 mm year^−1^), presents one rainy and one dry season [38, 41]. The mean rainfall and temperature of Cameroon and neighbours countries from 1985 to 2006 shows higher rainfall intensity in the western and coastal part of the country and increasing mean temperature moving north towards the Soudano-Sahelian zone (Fig. 1).

### Malaria data

**Fig. 2:**
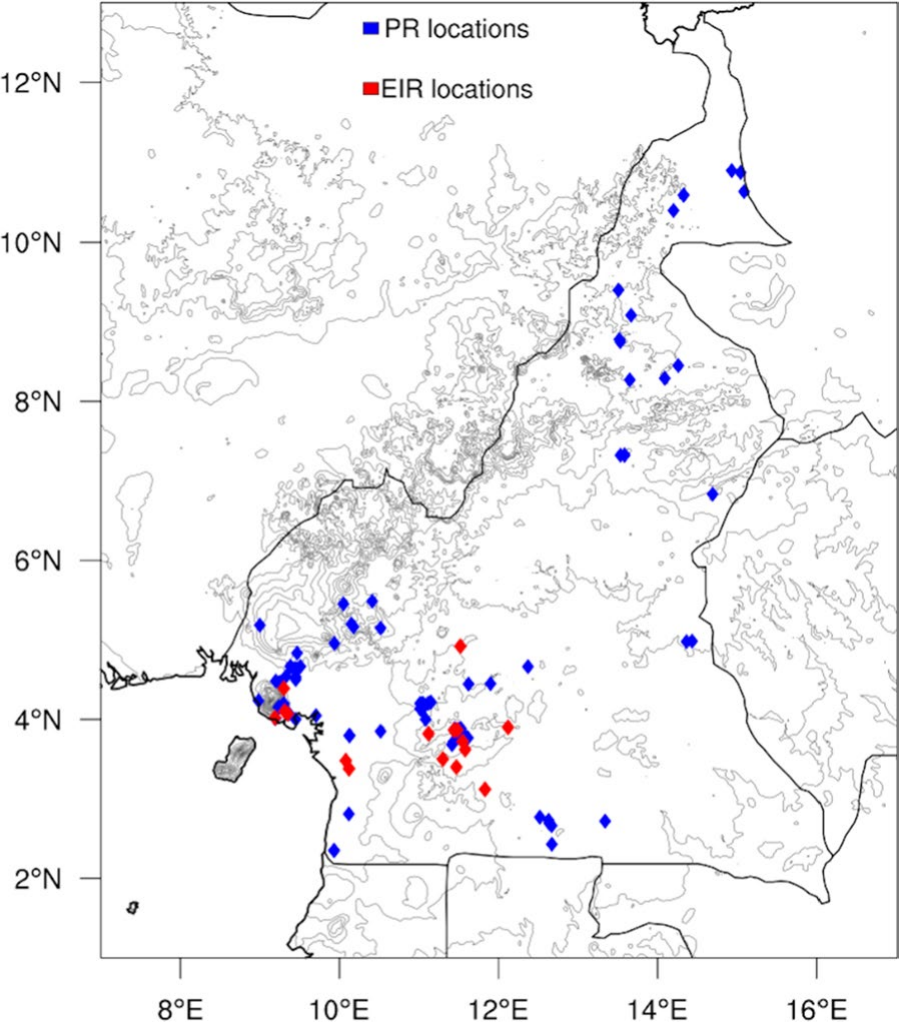
Map highlighting all the studies locations

Two malaria indicators are used in this study. The parasite ratio (PR) expresses the pro-portion of individuals infected at a given point in time [42]. A publicly available database of parasite ratio is obtained from the Malaria Atlas Project (MAP) programme [43]. The public PR database consists of data collected by individuals researchers or organizations and published in literature, which were collected within the MAP programme. Since there is no continuous measurement of PR, the available PR data with georeferenced coordinates are used. The location of the PR surveys is given in Fig. 2, which shows that the majority of surveys are located in the west or the far north, ant east of the country. In total, 103 surveys are used, with a total of 18,011 people tested in these surveys, with the survey dates ranging from 1985 to 2006.

All database entries have been quality controlled in terms of data collection methodology and geographical location to ensure continuity across the 20-year collection period. In addition to climate, population density and vicinity to water, many other factors may influence malaria transmission such as socioeconomic conditions, conflict, breakdown in health services, population movements and interventions, which are challenging to account for, not least due to lack of availability of data. As long as these factors are not correlated with spatial or temporal variability of climate, they will act as a form of noise in the analysis, increasing scatter in the climate-malaria relationships, but not obscuring them completely if climate is a significant driver of malaria variability. This is also the case for data inaccuracies and uncertainties in both the climate due to instrument error and sampling uncertainty [44] and health records. One complication might be if these facts lead to slow trends over the period, but this would most likely be associated with ramping up of interventions (climate trends are captured in the analysis) and this period predates the large-scale up of interventions that occurred in Cameroon that could confound the climate-malaria relationship. In addition, there have been entomological studies but none found changes is vector distribution during this period, and we assume that such changes would thus not have affected the mean climate-malaria relationships.

The second malaria indicator is the entomological inoculation rate (EIR), which measures the number of infected bites received per person for a given period of time [43], and as such is an indicator of the malaria transmission intensity. It is often calculated as the product of the human biting rate (HBR) and the sporozoite rate. HBR represents the number of bites per person per day, while the sporozoite rate is the fraction of vector mosquitoes that are infectious [45]. A new database of monthly EIR values has been constructed from various sources for all Africa by Yamba et al. [24], with the emphasis on long term field studies lasting at least a year in order to be able to study the seasonality of malaria transmission. For Cameroon, the database has recorded 16 sites with validated data presented in the following Table 1.

**Table 1:** Sites of EIR data points used in Cameroon

Site	Location	Longitude	Latitude	Period	References
1	Sanaga village	11.52	4.92	April 1989–March 1990	[46]
2	Mbebe	10.12	3.38	April 1989–March 1990	[47]
3	Nkol-bikok	11.52	3.87	March 1989–February 1990	[15]
4	Nkol-bisson	11.44	3.86	April 1989–March 1990	[15]
5	Limbe	9.19	4.02	August 2001–June 2002	[48]
6	Tiko	9.35	4.07	August 2001–June 2002	[48]
7	Likoko	9.3	4.39	October 2002–September 2003	[49]
8	Essuke-camp	9.31	4.1	October 2004–September 2005	[50]
9	Ebogo	11.47	3.4	April 1991–March 1992	[51]
10	Simbock	11.3	3.5	January 1999–December 1999	[52]
11	Koundou	12.12	3.9	June 1997–May 1998	[53]
12	Ekombite	11.83	3.12	January 2007–December 2007	[54]
13	Nsimalen-Ekoko	12.12	3.82	April 1991–March 1992	[55]
14	Nsimalen-Nkol-mefou	11.58	3.62	April 1991–March 1992	[55]
15	Nsimalen-3	11.55	3.72	April 1991–March 1992	[55]
16	Ndogpassi	10.08	3.48	January 2011–December 2011	[56]

The rarity of long-term, continuous monthly EIR records that allow the analysis of seasonality, necessitates the use of data from 30 years ago, but we reiterate that this has the advantage that recent upscaling of (sometimes seasonal) interventions does not obfuscate the analysis. The availability of data for only 2 years in time precludes any analysis of longer terms changes in seasonality that may be associated with climate warming which could potentially be significant [57]. The EIR data sites are highlighted on Fig. 2 below.

### VECTRI malaria model

The VECToR borne disease model of ICTP (VECTRI) is an open source gridded distributed dynamical model, that couples a biological model for the vector and parasite life cycles, to a simple compartmental Suceptible-Exposed-Infectious-Recovered (SEIR) representation of the disease progression in the human host. The model runs using daily time step temperature and rainfall data, but also accounts for the population density which is important for the calculation of daily biting rates [37]. The model incorporates several parameterizations schemes for larvae, adult vector and parasite development rates, which are both temperature sensitive, as are the larvae and adult vector daily survival. Larvae survival, especially in the early development stages, is also impacted negatively by intense precipitation through the inclusion of a flushing effect [58]. The model also allows for over-dispersive biting rates and incorporates a simple treatment of host immunity [59]. Another feature of the model is that it also includes a simple treatment of rain-driven pond formation and loss through evaporation and infiltration [29, 60, 61]. The model allows the user to specify a permanent water breeding fraction but this is not used in the experiments reported here. VECTRI simulates several parameters that help in assessing malaria incidence. Among them are the parasite ratio and entomological inoculation rate.

In this study, the model is integrated for 22 years (1985–2006) with a 3-year spin-up period at 0.03° × 0.03° resolution. Mean daily precipitation data are obtained from Famine Early Warning Systems Network ARC vesion 2 (FEWS-ARC2) [62], available at a spatial resolution of 0.1° × 0.1°. The daily gridded 2 m temperature data is taken from the ECMWF ERA-Interim reanalysis data at 0.75° × 0.75° spatial resolution [63], which are then statistically downscaled to the model resolution assuming a lapse rate of 6.5 K km^−1^ to adjust to the high resolution topography. For each grid cell point, population density is obtained from AFRIPOP [64], again interpolated to the model resolution using conservative remapping. AFRIPOP database links informations on contemporary census data across Africa using geographical longitude and latitude position points. After the integration is complete, the nearest grid cell to each field survey location is extracted for comparison. When the comparison to climate variables is made, for each field survey of PR, the average rainfall and temperature from the preceding 2 months are used, in order to account for the observed lag of 1 to 2 months between malaria and rainfall and the fact that PR is a time-integrated and thus smoothed quantity that reflects climatic conditions over the preceding period [27]. For the time series analysis of EIR, comparisons are made directly to the time series of climate variables for the observed period. As the precise days of surveys were not usually available, only the month, then there is an uncertainty in the lag of 2 weeks.

## Results

### Parasite ratio evaluation

**Fig. 3:**
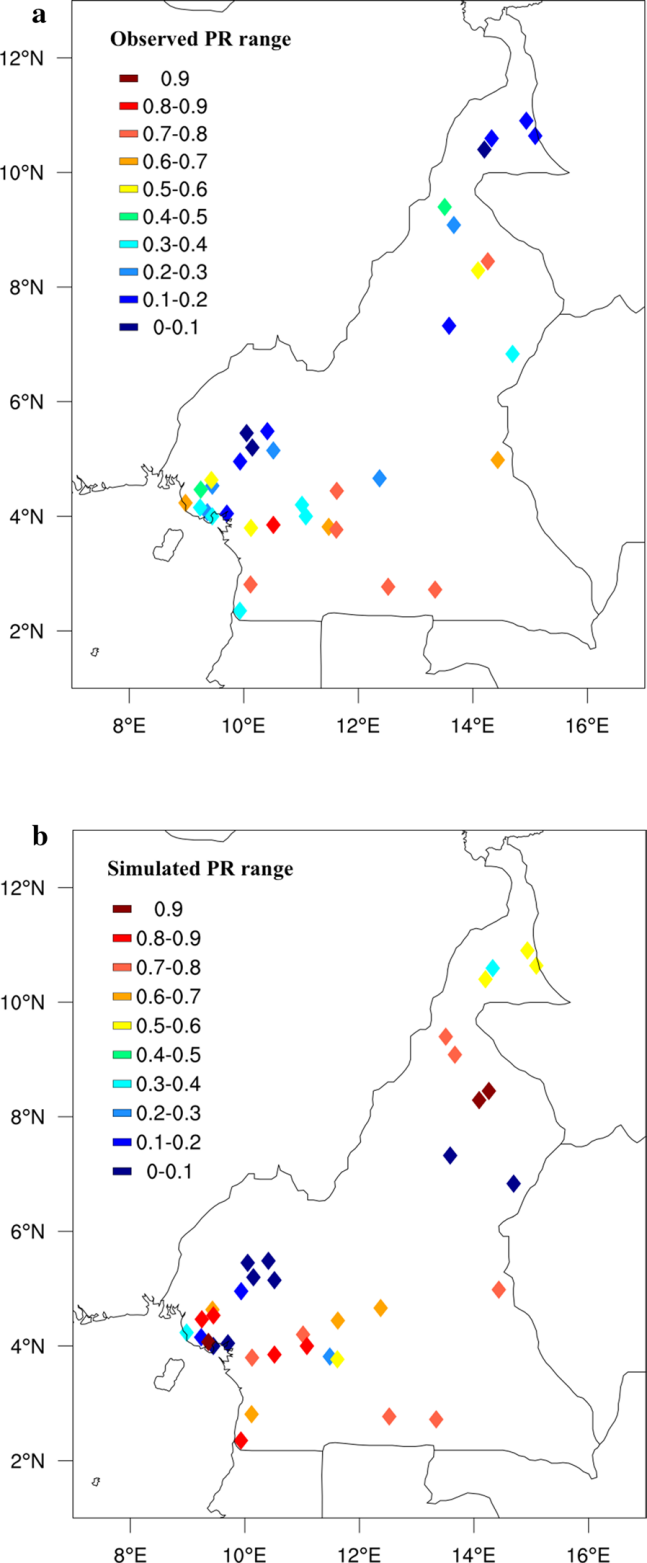
Observed (**a**) and simulated (**b**) monthly mean parasite ratio values for 36 sites in Cameroon. The PR values represent the average of all the points located within the same coordinates

The spatial maps of PR (Fig. 3) reveals a very heterogeneous landscape of malaria prevalence, particularly in the observed surveys, but also in the model. It should be recalled that the surveys are taken during different years and periods of the year, thus some of the variations are simply due to changes in the meteorology between survey times. Other factors such as interventions and population movements will also impact prevalence, but will not be reflected in the model simulations. Concerning the model, some regional biases stand out clearly. For example, the model produces PR values around 0.5 in the drier and warmer north east of the country, indicating conditions that are borderline between meso and hyperendemic, while the prevalence in the observations is far lower, indicating that the model is too sensitive to low rain rates.

**Fig. 4:**
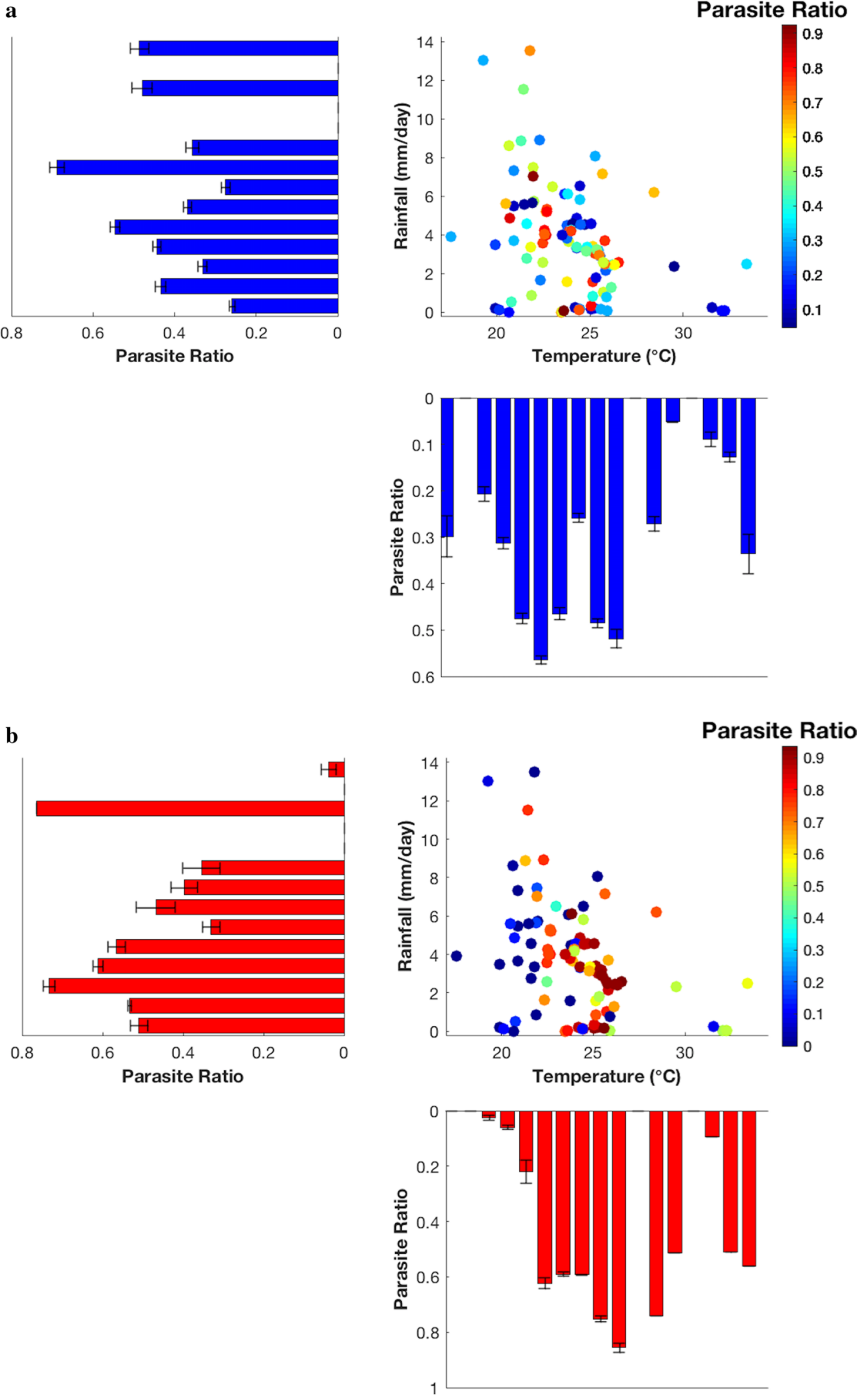
Observed and simulated parasite ratio, function of rainfall (mm/day) and temperature (°C) over Cameroon. Panels plots present how parasite ratio fluctuates with ranges of rainfall and temperature for observations and simulations. The bars indicate uncertainty, which for the observations is based on a statistical test on the proportion given the total number of people surveys in each bin. For the model the uncertainty measure is the standard deviation of the survey locations in each bin. **a** Observed data, **b** VECTRI model

To examine the mean relationship between PR and climate in more details, the survey and model results are divided into bins according to the two key climatic drivers of mean rainfall and temperature (Fig. 4). The field studies show the prevalence as measured by PR increases to a broad maximum from 22 to 26 °C. Prevalence then falls off but in still non-zero in the locations with mean temperatures above 30oC. The relationship with temperature is not smooth, as expected due to the fact that climate is only one of many external factors that impact the prevalence from location to location. The model produces a much sharper response to temperature, with low prevalence in the 18–21 °C range, and the peak transmission occurring around 26 °C with prevalence far higher than reported in the survey exceeding 80%. The response in PR to precipitation is more distinct in the model than observations. The observations reveal an increase in PR with increasing rainfall to a local maximum at 7 mm day^−1^. After the peak, PR decreases with increasing rainfall with the exception of the two bins of 11–13 mm day^−1^. The model instead peaks at a lower rainfall rate of 2 mm day^−1^, reducing thereafter, again with the exception of the second last, high rainfall bin.

**Fig. 5:**
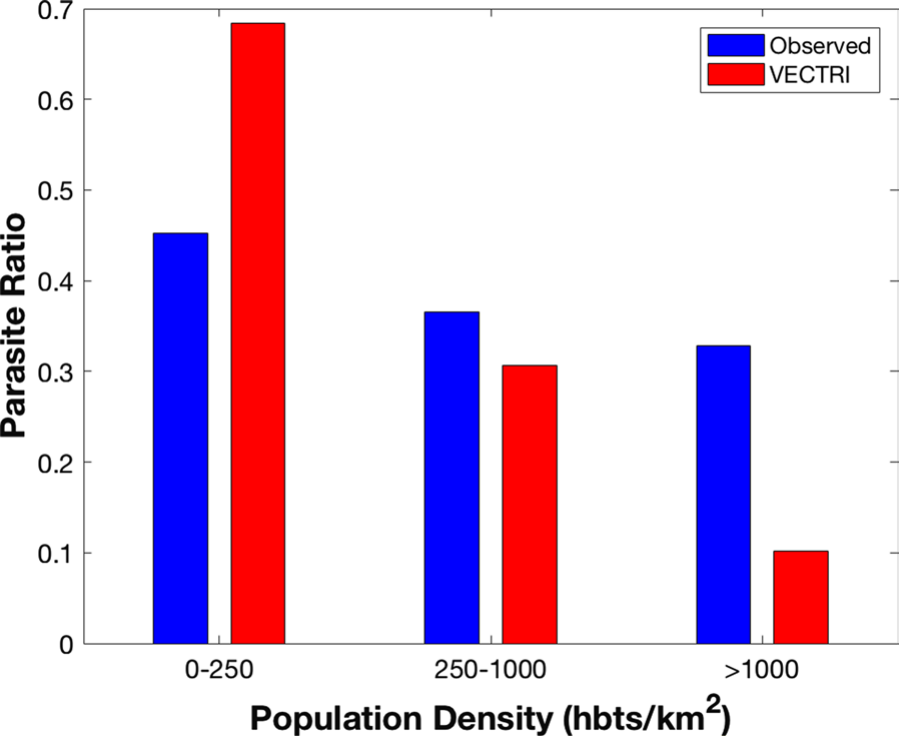
VECTRI and observed parasite ratio as a function of population density

The PR ratio is compared to population density assigned to three classes of rural (0 to 250 inhabitants per km^2^); peri-urban (250 to 1000 inhabitants per km^2^); and urban (> 1000 inhabitants per km^2^) according to Hay et al. [65]. The results are shown on Fig. 5. PR reduces with increasing population density, but with the relationship much stronger in the model relative to observations, a trait that was also observed by Tompkins et al. [37] when comparing EIR as a function of population to the survey data compiled by Kelly-Hope et al. [33]. Thus, the model appears to overestimate malaria prevalence in rural locations and underestimate it in urban centres.

### Seasonal EIR evaluation

**Fig. 6:**
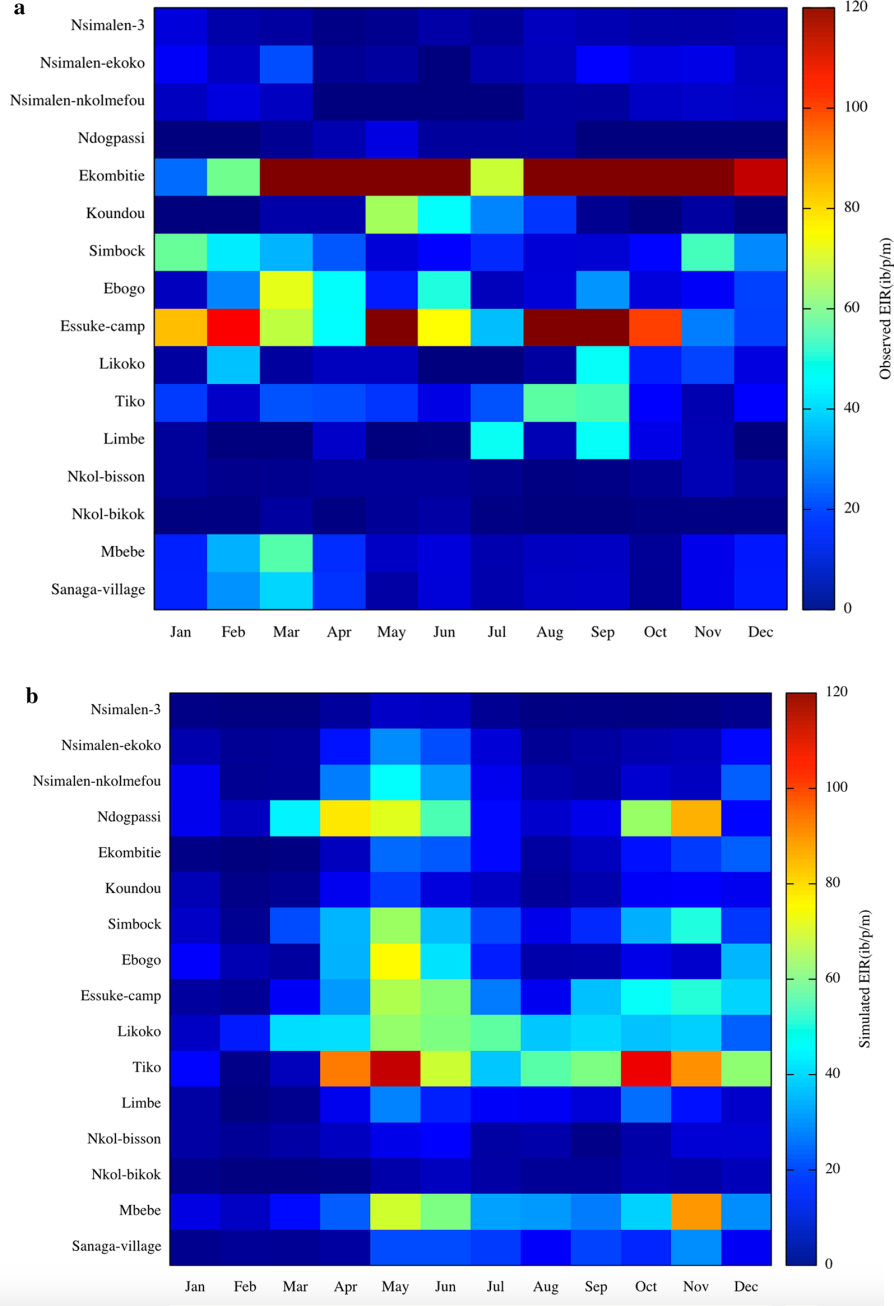

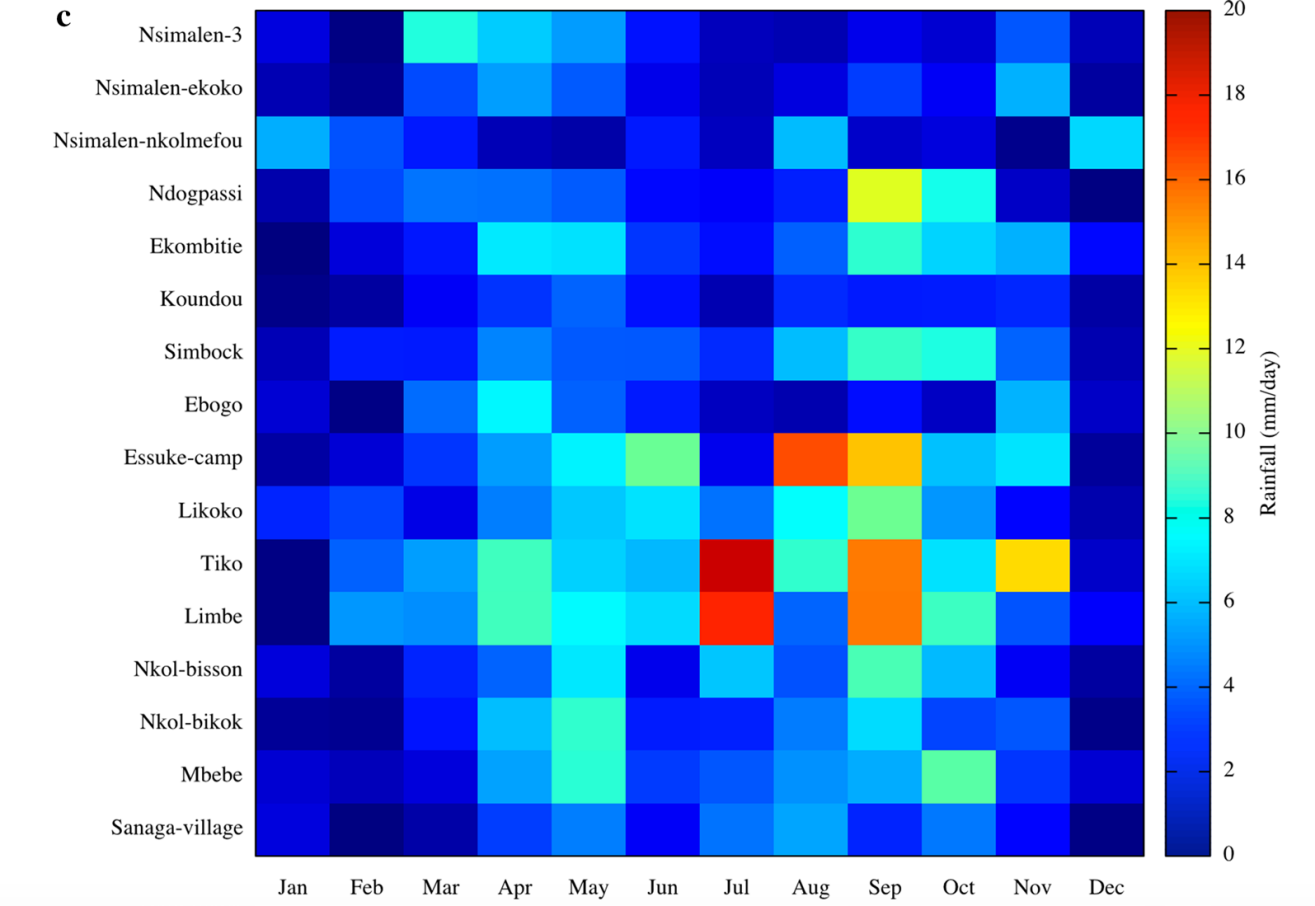
Observed (**a**), simulated (**b**) monthly mean entomological inoculation rate and **c** rainfall maps for the 16 EIR sites in Cameroon

The seasonal changes in monthly EIR for both model and observations during the study period for the sixteen locations as well as rainfall are presented in Fig. 6. The EIR in the model follows the patterns in rainfall in the studies locations with EIR lagging rainfall peaks by 1 to 2 months in each case. It is also the case for the survey data except in Ekombitie where the value are higher all year long. In certain locations like Sanaga village, Mbebe or Simbock, EIR seasonality is reversed, with peaks EIR values occurring during the relatively dry periods.

## Discussion

The temperature and rainfall sensitivity of the prevalence data is broadly in line with earlier works [66–68]. Favourable temperature ranges that support *Plasmodium falciparum* transmission via *Anopheles* species, is generally between 18 and 33 °C [69]. Simple models of the temperature impact on the proportion of female adult vectors surviving long enough for the parasite to complete the sporogonic cycle and permit transmission suggest that, transmission should peak at temperatures of approximately 28 to 32 °C [70]. Although these calculations are sensitive to the form of the adult mortality curve used and the temperature relationship with malaria remains poorly constrained. More recently suggestions have been made that, accounting for the temperature sensitivity of the vector larvae stages, results in a cooler peak temperature of around 25 °C [19]. Analysis of malaria indicators in Uganda and Rwanda reveals the peaks of malaria transmission occurring at 28 °C and 26 °C, respectively [71]. In the Zomba district in Malawi, a study found that malaria spread is at peak when temperature is at 24 °C [72]; while in the whole country cases monotonically increased with temperature to the maximum temperature sampled of 28 °C [12]. In Cameroon, the analysis reveals that the prevalence measured in surveys is maximum in the 22 to 26 °C range, although there is a gap in the survey sampling in the 27 to 31 °C range, and a warmer peak temperature cannot be precluded. The model similarly produces peak PR at 26 °C, in approximate agreement with the survey data and previous work.

The precipitation relationship is more complex, with PR maximized in survey data at 7 mm day^−1^. Usually moderate rainfall events are suitable for immature mosquitoes to complete the aquatic development stage, and emerge as adults [58]. Intense rains may cause flooding and flush out larvae from the habitats leading to a decrease in mosquito density [58, 73]. The survey data appears to be in good agreement with previous studies. In Botswana, cases peaked a rainfall rate of approximately 4 mm day^−1^, in Malawi the peak occurred at a high value of just over 6 mm day^−1^ [12] while in Uganda and Rwanda, highest cases numbers are associated with rainfall between 4 to 6 mm day^−1^ and 4 to 8 mm day^−1^, respectively [71].

No model will be able to reproduce such prevalence survey data perfectly, a model is necessarily a gross-simplification of reality. Even considering the climate-sensitive life-cycle processes that are accounted for, the model parameters are spatially and temporally homogeneous. For example, the hydrological parameters that determine the pond creation and subsequent loss through evaporation and infiltration are spatially constant, the temperature offset of breeding sites relative to the air temperature also. Moreover, many processes and factors that affect prevalence are not accounted for at all in the model, population movements are neglected, as are those of the vectors, no information on interventions is used, and the model for transmission in the host is extremely simple, neglecting superinfection and incorporating a very simple treatment of immunity. It could be argued that the data is not available to improve many of these aspects. That said, it is encouraging that the model at least manages to reproduce the underlying climate sensitivities revealed in the survey data.

Concerning the population sensitivity, PR in the survey data reduces as population density increases. This agrees with previous work [74], for instance, in Burkina Faso epidemiological profiles and clinical malaria transmission patterns tend to be high in rural compared to urban environments [24]. A review of entomological studies conducted across sub-Saharan Africa countries demonstrated that the higher number of annual *Plasmodium falciparum* EIR were reported in rural populations, where population density < 100 inhabitants per km^2^. However,

low EIR were measured in urban areas where population density > 1000 inhabitants per km^2^ [33]. This sensitivity is also apparent in the model, but the model appears to exaggerate the effect, tending to be higher relative to observations for rural settings, while under predicting PR in urban centres. For example, one survey was conducted in central Yaoundé by Quakyi et al. [75], with a prevalence of 0.5 to 0.6 revealed in the sampled population of 231 people. The population density in this location exceeds 9000 people km^−2^ and at such high densities the model fails to sustain transmission. One key process in such central urban locations is likely to be population movements, neglected in the model at present, with many of the cases likely to be imported. Other factors also impacts differences between rural and urban areas which are challenging to include in the model, for example, urban zones are associated with low transmission due to factors such as limited availability of breeding sites, improved environmental conditions, easy access to control interventions, housing types and among others [76]. For instance, Cameroon National Malaria Control Programme reported that bed nets are more used in urban than rural zones [77]. Most of these latter social and environmental impacts would act to increase disparities between rural and urban transmission, thus the crucial importance of mobility cannot be overlooked. In addition, the fact that the model neglects superinfection will also act to exaggerate the population density impact. In the model’s simple SEIR approach, once an infective bite results in successful transmission event, the host moves to an exposed state. The impact of large inoculations of multiple strains when many infectious bites are recorded is not included, thus that individuals enhanced capacity to further transmit the disease is neglected. This would lead to the model overestimating the population dilution effect.

In the survey data for the 16 EIR-sites, the EIR closely follows the seasonality of rainfall with a lag of approximately 1 month. The EIR maximizes in April, May and June while the second peak is observed in October, November and December. The observed seasonal variability of EIR agrees with variability in reported malaria cases, with high case numbers observed during and after rainy seasons [77]. In Nkoteng for example, Cohuet et al. [78] showed that malaria transmission intensity reaches its peak in April during the rainy season. In a related study in Niete (South Cameroon), Bigoga et al. [79] found a lower EIR during dry season (1.09 ibp^−1^n^−1^) compared to rainy season (2.3 ibp^−1^n^−1^). Similarly, comparing Simbock and Etoa districts, Quakyi et al. [75] found similar difference between rainy and dry seasons but a high disparity was observed for Etoa. They measured 1.9 ibp^−1^n^−1^ and 1.2 ibp^−1^n^−1^ for wet and dry seasons, respectively for Simbock and 2.4 ibp^−1^n^−1^ and 0.4 ibp^−1^n^−1^ for Etoa during the wet and dry season, respectively.

**Fig. 7:**
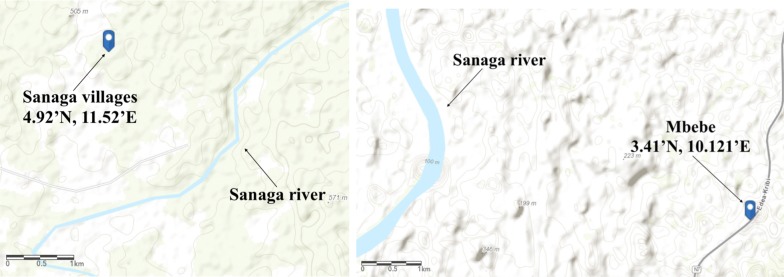
Sanaga villages and Mbebe locations, situated at the vicinity of the Sanaga river

The survey data for EIR in Sanaga villages, Mbebe, and Simbock contrasts strongly, and produces a seasonality of EIR which appears to be completely out of phase with the rainfall, with EIR at a maximum during the dry season, precisely January to March (for Sanaga villages and Mbebe) and (for Simbock), behaviour that VECTRI was unable to capture. One possible explanation for this disparity could be linked to their geographical situation and local hydrology. Simbock is located at about 100 m from the Mefou river creating a permanent swamp [52], while Sanaga villages and Mbebe are situated in the vicinity of the Sanaga river as presented on Fig. 7.

Rivers can and do support vectors at ponds formed at their edges, in particular *An. funestus*, and indeed the forested locations typical of these sites have identified *Anopheles nili*, *An. gambiae* and *An. funestus* as key malaria vectors [47]. *Anopheles nili* usually breeds among the grass on the edges of the river and can be a key driver of malaria transmission in such environments [46]. However, when such river systems are not managed, their impact on breeding sites can sometimes be enhanced during the dry season when flow is restricted and a large increase in the availability of standing pools can occur, constituting a proliferation of ideal breeding sites for *Anopheles* vectors [32, 80, 81]. The Sanaga river particularly undergoes a strong seasonal cycle in discharge, with flow at a minimum in February to April, with just a small fraction of the peak discharge during these months [82]. Thus, it seems in Sanaga villages and Mbebe, peak in malaria is associated with the minimum in the Sanaga river flow, and an enhancement in ponding. As this version of VECTRI does not account for permanent breeding site associated with river systems, with enhanced ponding in low flow periods, it is not able to reproduce the seasonal cycle in EIR here.

## Conclusion

The relation between climate and two common malaria indicators of parasite ratio (PR) and entomological inoculation rate (EIR) were examined in Cameroon, using a comprehensive of survey data for PR and others surveys for EIR that enabled the seasonality of transmission intensity to be examined. While many factors can impact malaria transmission, the established boards relationships of malaria climate drivers were apparent in the.

survey data, with PR increasing with temperature until a peak within 22–26 °C and thereafter reducing, with peak prevalence occurring at rainfall rates at 7 mm day^−1^. The analysis also confirmed previous research regarding the impact of population density, with PR higher in rural areas relative to urban areas.

The seasonal cycle of the EIR revealed very contrasting behaviour between peri-urban sites, and rural sites situated closely by the Sanaga or the Mefou river. In the peri-urban sites, the EIR seasonality closes follows that of the rainfall, with maxima lagging rainfall peaks by 1 to 2 months. Instead, in rural ones the EIR seasonality is out of phase with rainfall and peaks in March–April when the Sanaga discharge is at its annual minimum, indicating a strong role for the pooling in the river-bed in providing seasonal breeding sites for vectors.

The malaria model is able to reproduce some of these broad traits of the malaria transmission indicators, with a similar relationship between PR and the mean temperatures, while the prevalence peaks at a lower value of rainfall. The model also reproduces the reduction in PR with increasing population. In general, the model produces a too high contrast between areas of high and low transmission relative to the surveys, indicating that a mixing effect, most likely in the form of human migration patterns is lacking in the model in addition to the lack of superinfection. The model is able to reproduce the seasonality of the EIR only in the locations where transmission intensity closely follows temporary breeding sites resulting directly from rainfall, and it cannot produce the dry season peak in the locations near the Sanaga river where breeding sites occur due to low rain flow and Mefou river as well. Thus, while there are numerous simplifications and neglected processes in the model, it would appear that the coupling of the malaria transmission scheme with a model to represent human population movements [83], and the improved representation of breeding sites due to semi-permanent features such as rivers, lakes and dams should be a priority. In general, the model produces infectious biting rates that exceed those observed, and it is likely that, if the model is to be used to aid operational decisions in Cameroon, the use of machine learning techniques to calibrate the model parameters more effectively will be required, such as that recently introduced in Tompkins et al. [44].

## Data Availability

The datasets used and/or analysed during the current study are available from the corresponding author on reasonable request.

## Abbreviations

VECTRI: VECTor borne disease community model of the International Centre for Theoretical Physics, TRIeste
ICTP: International Centre for Theoretical Physics
PR: parasite ratio
EIR: entomological inoculation rate
MAP: Malaria Atlas Project
ibp^−1^ m^−1^: infective bites per person, per month
ibp^−1^ n^−1^: infective bites per person, per night
